# Cultural adaptation and validation of the “Pregnancy Physical Activity Questionnaire” for the Portuguese population

**DOI:** 10.1371/journal.pone.0279124

**Published:** 2023-01-10

**Authors:** Paula Clara Santos, Leonardo Y. S. Maciel, Sandra Abreu, Ana Rita Mesquita, Cristina Carvalho Mesquita, Sofia Lopes, Jorge Mota

**Affiliations:** 1 Department of Physiotherapy, School of Health, Polytechnic of Porto, Porto, Portugal; 2 Research Centre in Physical Activity, Health and Leisure (CIAFEL)—Faculty of Sport—University of Porto (FADEUP) and Laboratory for Integrative and Translational Research in Population Health (ITR), Porto, Portugal; 3 Center for Research in Rehabilitation, School of Health, Polytechnic of Porto, Porto, Portugal; 4 Department of Physical Therapy, Federal University of Sergipe, Lagarto, SE, Brazil; 5 Postgraduate Nursing Program, Federal University of Sergipe, Aracaju, SE, Brazil; 6 Department of Physiotherapy, Tâmega e Sousa Higher School of Health Technologies, Polytechnic Health Institute of the North, (CESPU), Gandra, Paredes, Portugal; Universiti Malaya, MALAYSIA

## Abstract

**Background:**

The lack of instruments to assess the level of physical activity in pregnant women, led to the development of the PPAQ (Pregnancy Physical Activity Questionnaire), a self-administered questionnaire, which has already been translated in several countries and has already been used in several studies.

**Aim(s):**

Translate and adapt the PPAQ into Portuguese and test its reliability and validity.

**Methods:**

An analytical observational study was carried out. Linguistic and semantic equivalence was performed through translation and back-translation and content validity was tested by a panel of experts. To test reliability, a test-retest was performed on a sample of 184 pregnant women, with an interval of 7 days and the ICC was used. To test the criterion validity, Pearson’s correlation coefficient (r) was used between the PPAQ and the accelerometer, in a sample of 226 pregnant women.

**Findings:**

The questionnaire was considered comprehensive. The ICC values of Reliability were: total score (0.77); sedentary activities (0.87); light-intensity activities (0.76); moderate-intensity activities (0.76); vigorous-intensity activities (0.70). For criterion validity was obtained a coefficient correlation of r = -0.030, considered weak and negative, for total activity.

**Discussion:**

This study describes the translation and validation process of the PPAQ questionnaire from English to Portuguese. The final version of the PPAQ was considered as a valid instrument in terms of content to measure physical activity and was referred to as being simple to apply and easy to understand.

**Conclusion:**

The PPAQ has content validity, excellent reliability and weak criterion validity, as in the original version.

## Introduction

Physical Activity (PA) is defined as “any bodily movement produced by skeletal muscles that results in energy expenditure” [1]. A large body of data has demonstrated the importance of PA during pregnancy [2–10] and various organizations recommend PA during pregnancy such as the United States Department of Health & Human Services (USDHHS), the World Health Organization (WHO) [11], the American College of Obstetricians and Gynecologists (ACOG) [2] and the Centers for Disease Control and Prevention/American College of Sports Medicine (CDC/ACSM) [12].

The practice of regular PA during pregnancy generates positive effects on the health of women and babies [8], such as gestational weight control, reduction of gestational diabetes (reduction in maternal glucose and insulin levels and increase in maternal insulin sensitivity), and pre-eclampsia, as well as the reduction in the number of cesarean deliveries [2–4, 6–8]. It also improves sleep quality, well-being, and self-esteem [3, 7], promoting a better quality of life and good physical and mental health [6].

Henceforth, designing tools to monitor and assess PA during pregnancy is essential; at this point, valid and reliable instruments are necessary. Questionnaires are a simple assessment tool: they have the ability to be self-administered, non-invasive, and do not require expensive and complicated technical equipment [8]. There are several questionnaires to assess PA due to the expansion that this topic has in terms of public health and research, such as the IPAQ (The International Physical Activity Questionnaire), the KPAS (Kaiser Physical Activity Survey) and the PIN3 (Pregnancy Infection and Nutrition 3) [13, 14]. In spite of that, few are appropriate for the specific condition of the pregnancy.

The “Pregnancy Physical Activity Questionnaire” (PPAQ) [15] is a self-administered questionnaire that was developed to measure and assess levels of PA among pregnant women. It measures the frequency and duration of different types of activities and provides an intensity value for each activity [6, 8, 15]. It has been translated and validated for several countries, namely: Vietnam [16], Brazil [17, 18], Turkey [19, 20], Spain [21], Poland [22], Japan [23], China [24], France [25], Arabia [26], South Korea [27] and Denmark [28], as it has already been used in numerous clinical studies [5, 6, 29, 30]. Taken altogether, that allows the collection of reliable data and facilitates the comparison and discussion of the results of different studies, including studies at an international level [31, 32].

For the cultural adaptation of an instrument, according to the European Group on Health Outcomes (ERGHO), both an evaluation of the linguistic or semantic equivalence and an evaluation of the psychometric properties following established guidelines and standards for cultural adaptation are necessary [33]. The psychometric analysis comprises the assessment of the quality of an instrument, based on proof of reliability and validity [31, 32].

As mentioned, there is a Brazilian Portuguese version of the PPAQ [17, 18] validated for the Brazilian population. However, given that they are two different cultures, there is no guarantee that this version will demonstrate the necessary equivalence to the original English version or even to each other.

Therefore, in order to allow the Portuguese population access to this important questionnaire and allow Portuguese science to produce multicentric research with other countries, more reliably in the future, there was a need to culturally adapt and validate the questionnaire for Portuguese pregnant women. Thus, the aim of this study was to translate and cross-culturally adapt the PPAQ into Portuguese and test its reliability and validity.

## Methods

### Study design

An analytical observational study was carried out, with those who presented any of these exclusion criteria being considered ineligible for the study: insulin-dependent diabetes, hypertension or heart disease that required medication, chronic kidney disease, and those who were younger than 16 or older than 40 years old [15, 16].

#### Phases

This study was carried out in different stages in which 3 samples were used.

#### Content validity stage

We used a convenience sample composed of 6 pregnant women (two who were in the 1st trimester, two who were in the 2nd trimester, and the other two who were in the 3rd trimester of pregnancy), with characteristics common to the target population of the study.

#### Criterion validity step

A total of 273 women were invited, a sample based on previous work [17, 26, 34–36], who lived in the district of Porto and performed the ultrasound examination at the University Hospital Center of São João (CHUSJ), Porto. Two hundred and fifty-eight women agreed to participate in the study, and of these, 226 women were considered as legible and constituted sample 2.

#### Reliability step

Two hundred and four pregnant women were invited, who were being supervised and followed up in parenting consultations at the *Unidade local de Saúde do Alto Minho (ULSAM)* We obtained a positive response from 197 women, of which 184 were considered as legible and thus constituted the sample.

#### Instruments

*Pregnancy Physical Activity Questionnaire*. The PPAQ is a self-administered questionnaire for pregnant women, which takes approximately 10 minutes to complete. It is a semi-quantitative questionnaire, which includes questions addressed to the participants about how much time is spent on 32 daily activities, including domestic, occupational, and sports/exercise activities. Participants, for each activity, are invited to select the category that best approximates the amount of time spent in that activity, per day or per week, during the gestational trimester in which they are found [15, 37].

In order to calculate the energy expenditure, using the PPAQ, the instructions of the authors of the original questionnaire were used. The time spent on each activity was multiplied by its intensity, obtaining a measure of weekly energy expenditure (MET hours. week1 or MET.h. wk^-1^), for each activity. In order to determine the intensity, the specific metabolic equivalent (MET) was assigned to each activity [6, 15, 37], according to the “Compendium of physical activities: an update of activity codes and MET intensities” [38].

The activities referred to in the PPAQ are classified according to intensity: sedentary (<1.5 METs), light (1.5 ≤ 3.0 METs), moderate (3.0 ≤ 6.0 METs) or vigorous (>6.0 METs) [6, 15]. To calculate the total weekly energy expenditure and that relating to different intensities and types of physical activity, the formulas shown in Table 1 were used.

**Table 1:** Formulas for weekly energy calculation using the PPAQ.

	Activity	Formula
	**Total activity**	Sum (duration x intensity) of questions 4 to 36;
**Intensity**	**Sedentary activity**	Sum (duration x intensity) of questions 11, 12, 13, 22 e 32
	**Light activity**	Sum (duration x intensity) of questions 4, 5, 7, 15, 16, 17, 18, 20, 34, and 30, 31 if ≤2,9 METs*
	**Moderate activity**	Sum (duration x intensity) of questions 6, 8, 9, 10, 14, 19, 21, 23, 24, 27, 28, 29, 33, 35, 36, and 30, 31 if ≥3,0 and ≤6 METs*
	**Vigorous activity**	Sum (duration x intensity) of questions 25, 26, and 30, 31 if >6,0 METs*
**Type**	**Domestic activity**	Sum (duration x intensity) of questions 4, 5, 6, 7, 8, 9, 10, 15, 16, 17, 18, e 19
	**Occupational activity**	Sum (duration x intensity) of questions 32, 33, 34, 35, e 36
	**sport activity/exercise**	Sum (duration x intensity) of questions 23, 24, 25, 26, 27, 28, 29, 30 e 31

*MET = metabolic equivalent

*Sociodemographic questionnaire*. The sociodemographic questionnaire included questions related to age, height, pre-pregnancy weight, marital status, education, and professional status.

*Accelerometer*. The accelerometer used was the ActiGraph GT3X (ActiLife v6.1.2, Actigraph, LLC, United States). This accelerometer was developed to detect an acceleration magnitude between 0.05 and 2.00 G and with a frequency response between 0.25 and 2.50 Hz, in order to differentiate normal human movement from other sources of acceleration, such as for example, riding in a car.

Accelerometers that recorded at least 480 minutes of daily use, at least 2 days a week and 1 day on weekend, were considered valid. The data obtained by the accelerometer, expressed in counts, were downloaded to a laptop and later analyzed by a program provided by the manufacturer, the Actilife software. The protocols used were based on the best recommendations for practices with accelerometers [39].

Activity levels were expressed in counts/min and for their classification, cut-points from the Freedson, Melanson, and Sirard (1998) protocol were used: <100 counts/min (sedentary activity); 100–1,951 counts/min (light-intensity activity); 1,952–5,724 counts/min (moderate-intensity activity); >5,724 counts/min (vigorous-intensity activity) [40].

*Anthropometric measurements*. Height was measured using the Harpenden Portable Stadiometer (Holtain Ltd, UK) and values were recorded in meters.

Participants were categorized according to the pre-pregnancy BMI, according to the World Health Organization [11], as follows: low weight (<18.5 kg/m2), normal range (18.5–24.9 kg/m2), overweight (25.0–29.9 kg/m2) and obesity (≥30.0 kg/m2). Pre-pregnancy weight was self-reported by the woman.

### Procedures

#### Translation and cultural adaptation

To use the instrument and make the cultural and linguistic adaptation of the PPAQ questionnaire to Portuguese, authorization was requested from the first author of the questionnaire, Lisa Chasan-Taber, and that was granted.

After obtaining permission, linguistic or semantic equivalence was carried out through the translation of the questionnaire. The English version of the PPAQ was independently translated into Portuguese by two official translators whose mother language is Portuguese. After obtaining two versions (VA and VB), these were worked on to produce a common version (V AB). This synthesis was carried out by a panel consisting of translators (A and B) and three specialists in the field of PA and Women’s health (JM, PCS, RS). This joint work aimed to resolve discrepancies between the independent original translations and obtain a common translation, consisting of relevant items, in order to assess PA during pregnancy. Subsequently, a back translation (VC) was carried out, which consists of translating the questionnaire produced in Portuguese into English (the original language), carried out by two official professional translators whose mother language is English and who speak fluent Portuguese. These translators were blind and had no prior knowledge of the questionnaire. Finally, a review of syntactic and grammatical errors was performed, and a pre-final version of the questionnaire (pre-PPAQ) was developed by consensus.

The final Portuguese version of the PPAQ was only defined after the application of the first stage of our study, where the pre-test of the questionnaire was applied to 6 pregnant women. They filled out a document where they were asked each question if the question was relevant and understandable.

#### Validity

Participants in sample 2 were invited to fill out the PPAQ and the sociodemographic questionnaire and, on the same day, began to use the accelerometer to objectively assess the PA levels. This was placed over the anterosuperior iliac spine, on the right side, held by an elastic strap (adjustable belt), and was used daily to obtain more detailed information about the PA of pregnant women for 7 consecutive days. The women also received a form where they recorded the activities carried out throughout the day, as well as the removal of the accelerometer, in situations such as swimming, taking a shower, or even during periods of rest.

The accelerometers and the form were returned by the women, in a pre-paid padded envelope and with the printed sender provided by the investigators. If the return of the envelope did not occur within a maximum period of 2 days after the agreed period, the women were contacted via SMS to make the return.

#### Reliability

To determine the instrument’s reliability, a test-retest was performed. Initially, sample 1 participants were invited to fill out a first PPAQ questionnaire (PPAQ1) and a sociodemographic questionnaire. After 7 days [15, 16, 19, 20], they were asked to complete the PPAQ questionnaire (PPAQ 2) again.

The values were compared to check the reliability of the questionnaire in a general way (total activity) and in a more specific and detailed way, being divided according to the activity intensity: sedentary activity, light-intensity activity, moderate-intensity activity, and vigorous-intensity activity; regarding the type of activity, it was divided as follows: occupational, domestic and sports/exercise activities.

#### Ethical aspects

All participants were informed verbally and in writing about the objectives of this study, and after reading the document, they signed the informed consent form in accordance with the Declaration of Helsinki [41].

The study was approved by the Ethics Committee of the Hospital de São João (Reference No. 09988) and authorized by the ULSAM board of directors. It was conducted in accordance with the World Medical Association´s Helsinki Declaration for Human Studies.

#### Statistic

To carry out the statistical analysis, the IBM SPSS program, version 21.0, was used, with a significance level of 0.05 and a 95% confidence interval.

To test the normality of samples 1 and 2, the Kolmogorov-Smirnov test was used. Sample 1 did not follow normality and, therefore, non-parametric tests were used. Sample 2 followed normality and parametric tests were used [42].

The intraclass correlation coefficient, the standard error of measurement (SEM) and the Bland-Altman method were used to analyze reliability (sample 1). The ICC value was considered as “excellent” when ≥ 0.75, “good” when 0.4 ≤ ICC < 0.75 and “poor” when ICC < 0.4 [42].

The scatter plots (Bland-Altman) relate the means of the two questionnaires (PPAQ1+PPAQ2)/2, on the X axis, with the bias (difference between them), PPAQ2 –PPAQ1, on the Y axis, in addition to showing the limits of agreement (±2SD). This method allows evaluating the relationship of disagreements with the evaluated measures.

To assess validity (sample 2), the PPAQ means were compared with the accelerometer values using the T-test for paired samples, and the correlation of variables was determined using Pearson´s correlation coefficient (r). A weak correlation was considered if |r| was between 0 and 0.3, moderate if 0.3≤ |r| <0.6, strong if ≤0.6 |r| < 0.9 and very strong if |r| ≥0.9 [42].

#### Inclusivity in global research

Additional information regarding the ethical, cultural, and scientific considerations specific to inclusivity in global research is included in the S1 Appendix.

## Results

### Semantic equivalence and content validity

After the translation and back-translation of the instrument, the pre-final version of the PPAQ was tested, having been considered a simple and easy-to-understand instrument from the original version. Thus, a consensus was reached on the final translation of the instrument, as it did not present ambiguous concepts and was considered to be easy to read and understand.

At the meeting held with the committee of experts, some changes were suggested in the way the questions were prepared, in order to correct the semantics and make them easier to read. Thus, the changes were intended to improve the syntax and semantics of the questions. The changes can be consulted in Portuguese and English in S2 Appendix.

The pre-test carried out by the 6 participants revealed that the questionnaire was understandable and easy to complete, and they did not propose any type of change.

The final title of the questionnaire was: “Questionnaire on Physical Activity and Pregnancy”.

**Fig 1:**
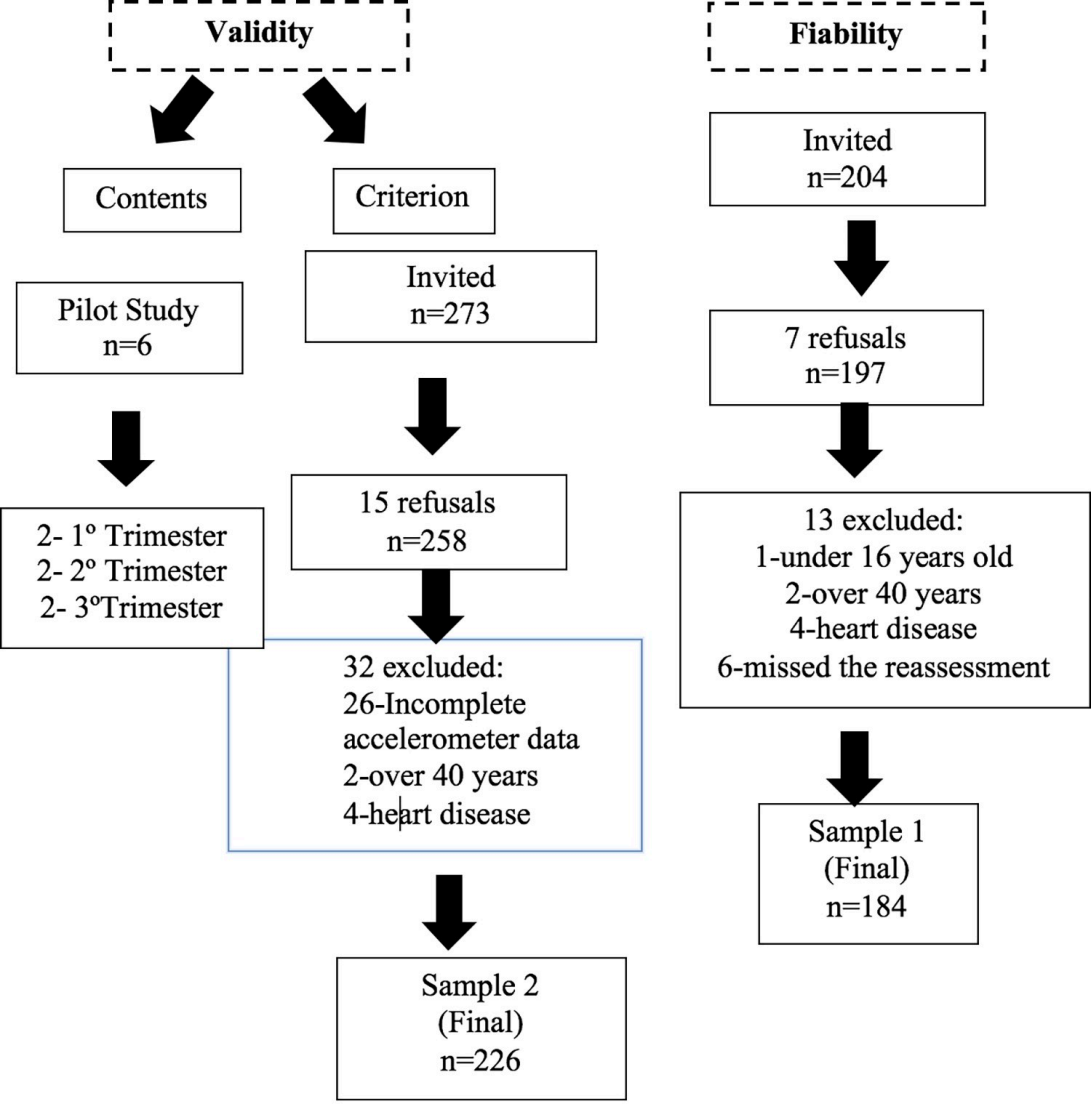
Flowchart of samples for content validity, criterion validity and reliability.

In this study, 226 pregnant women participated in the criterion validity sample and 184 in the instrument reliability calculation. In Fig 1, we can observe the flow of behavior of our sample at all stages (content validity, criterion validity, and reliability).

### Reliability

Table 2 represents the characterization of the sample that participated in the reliability analysis (test-retest) of the PPAQ questionnaire. Most women (59.8%) were in the 3rd trimester of pregnancy and 48.4% were aged between 30 and 35 years. Most women were married or living in a common-law relationship (75%) and 62.5% were employed.

**Table 2:** Sociodemographic data (n = 184) of sample 1, related to the PPAQ reliability test.

Characteristics			Characteristics		
	n	%		n	%
**Age groups (years) n = 184**			**Gestation trimester n = 184**		
18–23	9	4,9	1° Trimester	37	20,1
24–29	47	25,5	2° Trimester	37	20,1
30–35	89	48,4	3° Trimester	110	59,8
36–40	39	21,2			
**Pre-gestational BMI (kg/m** ^ **2** ^ **) n = 151**			**Marital status n = 160**		
Low weight	2	1,1	married or living in a common-law relationship	138	75
Normal weight	101	54,9	Single	21	11,4
Overweight	40	21,7	Separated/divorced	1	0,5
Obesity	8	4,3			
**Education (years) n = 160**			**Employment status n = 163**		
<5	3	1,6	Employee with contract	87	47,3
5–10	45	24,5	Employee without contract	28	15,2
[10–12]	58	31,5	Domestic	7	3,8
College course	51	27,7	Unemployed	31	16,8
Master’s degree	2	1,1	Student	2	1,1
Other	1	0,5	Other	8	4,3

The median values of weekly energy expenditure of the PPAQ1 were higher than the results obtained by the PPAQ2 in relation to the total activity (216.84 MET-h.wk^**-1**^ and 202.34 MET-h.wk^**-1**^, respectively) (Table 3). The results whose difference was greater refer to light-intensity activities and household activities, which were higher in the PPAQ1.

**Table 3:** Median of the PPAQ1 and PPAQ2 questionnaires (MET-h.wk^-1^) for total physical activity and for physical activity as a function of intensity and type (n = 184).

		PPAQ1 (MET-h.wk^-1^)			PPAQ2 (MET-h.wk^-1^)		
		P25	Median	P75	P25	Median	P75
	**Total physical activity**	153,79	216,84	259,51	142,65	202,34	259,64
**Physical activity**	**Intensity**						
	Sedentary (<1.5 METs)	24,89	46,73	81,90	26,82	50,49	79,40
	Light (1.5≤ 2.9 METs)	67,68	98,44	133,92	61,56	86,89	121,23
	Moderate (3.0–5.9 METs)	20,74	38,61	79,71	17,00	42,35	80,95
	Vigorous (≥6.0 METs)	0,00	0,00	1,63	0,00	0,00	1,63
	**Type**						
	Domestic	53,29	81,64	115,02	50,41	67,90	110,94
	Occupational	0,00	62,91	96,39	0,00	66,50	113,40
	Sport/exercise	2,40	6,18	10,31	2,44	6,05	10,56

PPAQ1 = 1st application of the Pregnancy Physical Activity Questionnaire; PPAQ2 = 2nd application of the Pregnancy Physical Activity Questionnaire; MET = metabolic equivalent; P25 = 25th percentile; P75 = 75th percentile

The floor effect matters (i.e., choose the lowest category). Most of these questions have more than 50% of the answers in the "none" category. We did not identify any with a ceiling effect.

In Table 4, we can see that the ICC values are considered excellent for all types of PA, except for vigorous activity and sports activity/exercise, which were good (0.70 and 0.72, respectively). The ICC obtained for the total PPAQ score (total activity) was 0.77. Better ICC results were obtained for sedentary activities (0.87) and for occupational activities (0.83).

**Table 4:** Intraclass Correlation Coefficient (ICC) and Standard Error of Measurement (SEM) between PPAQ1 and PPAQ2 (n = 184).

		ICC (95% CI)	SEM
	**Total Activity**	0,77 (0,70–0,82)	55,24
Physical activity	**Intensity**		
	Sedentary (< 1.5 METs)	0,87 (0,82–0,90)	12,52
	Light (1.5≤ 2.9 METs)	0,76 (0,69–0,81)	25,43
	Moderate (3.0–5.9 METs)	0,76 (0,69–0,81)	38,06
	Vigorous (≥ 6.0 METs)	0,70 (0,62–0,76)	1,43
	**Type**		
	Domestic	0,80 (0,73–0,84)	27,31
	occupational	0,83 (0,78–0,87)	34,03
	sport/exercise	0,72 (0,65–0,79)	3,97

ICC = Intraclass coefficient; SEM = Standard error of measurement; MET = metabolic equivalent of task

**Fig 2:**
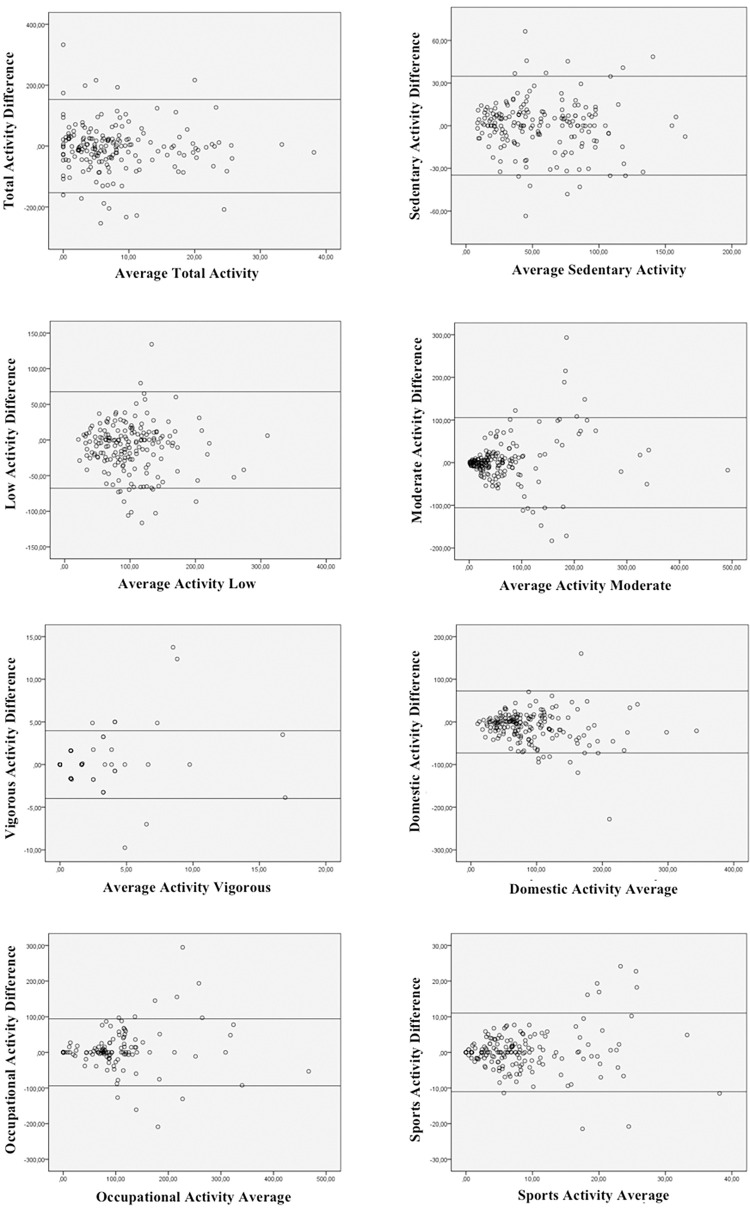
Dispersion graphs (Bland-Altman) for the results obtained by the PPAQ1 and PPAQ2, according to the intensity and type of physical activity.

The Bland-Altman graphs (Fig 2) show the agreement between PPAQ1 and PPAQ2 for total activity and for different intensities and types of physical activity. The graphs, represented in Fig 2, do not reflect any type of trend. Furthermore, there are few results that exceed the limits of agreement (±2SD).

### Validity

Table 5 shows the characterization of sample 2, which participated in the PPAQ validity test. Pregnant women in this study were mostly aged between 30 and 35 years (45.1%) and most were in the 1st and 2nd gestational trimesters (90.3%). Regarding marital status, 77.9% of the women were married or living in a common-law relationship and 74.4% of the participants were employed.

**Table 5:** Sociodemographic data (n = 226) of sample 2 related to the PPAQ validity test.

Characteristics	n	%	Characteristics	n	%
**Age groups (years) n = 226**			**Gestation trimester n = 226**		
18–23	27	11,9	1° Trimester	104	46,0
24–29	58	25,7	2° Trimester	100	44,3
30–35	102	45,1	3° Trimester	22	9,7
36–40	39	17,3			
			**Marital status n = 225**		
**Pre-gestational BMI (kg/m2) n = 226**			Married/ living in a common-law Relationship	176	77,9
Low weight	3	1,3	Single	38	16,8
Normal weight	125	55,3	Separated/divorced	11	4,9
Overweight	67	29,6			
Obesity	31	13,7	**Employment status n = 225**		
			Employee with contract	131	58,0
**Education (years) n = 225**			Employee without contract	37	16,4
<5	7	3,1	Domestic	2	0,9
5–10	73	32,3	Unemployed	44	19,5
[10–12]	75	33,2	Student	4	1,8
College course	66	29,2	Other	7	3,1
Master’s degree	4	1,8			
Other	0	0			

Table 6 shows Pearson’s r correlation values between the PPAQ and the accelerometer, regarding criterion validity. A weak correlation was obtained, with no statistically significant differences for all variables, except for light-intensity activities, in which the weak correlation was significant (r = 0.149; p = 0.025).

**Table 6:** PPAQ and accelerometer means and Pearson’s r correlation coefficient values between the PPAQ and the accelerometer (n = 226).

Physical activity	Average (SD) PPAQ (MET-h.wk-1)	Average (SD) Accelerometer (counts/min)	Mean of differences (SD)	*r*	p
**Total Activity**	260,62 (138,04)	1281,93 (88,11)	1021,31 (165,98)	-0,030	0,653
**Sedentary**	53,59 (33,18)	1154,84 (89,90)	1101,25 (93,27)	0,081	0,225
**Light**	109,80 (52,31)	80,51 (36,39)	-29,26 (59,11)	**0,149**	**0,025**
**Moderate**	96,07 (106,90)	31,06 (17,55)	-65,01 (108,28)	0,003	0,960
**Vigorous**	1,16 (3,32)	15,51 (57,93)	14,36 (57,67)	0,106	0,111

PPAQ = Pregnancy Physical Activity Questionnaire; SD—Standard Deviation

## Discussion

This study describes the translation and validation process of the PPAQ questionnaire from English to Portuguese. The final version of the PPAQ was considered as a valid instrument in terms of content to measure PA and was referred to as being simple to apply and easy to understand.

In terms of reliability, this study obtained, for the total activity, an ICC of 0.77, which is suggestive of excellent reliability. This result is like other studies carried out previously [15, 22, 24], in which the result obtained in the original study was 0.78. The validation study for the Spanish population had the highest ICC value of 0.90 and the validation study for the Polish population had the lowest ICC value of 0.75. The sample size varied, in the different studies, between 54 (original PPAQ validation study) and 109 (Spanish validation study) pregnant women [15, 16, 20–22]. The higher ICC values obtained (Spain and Vietnam) may be related to the fact that the two applications of the questionnaire are face-to-face [16, 21], which may lead to a social desirability bias. Also in this study, vigorous-intensity activities and sports activities had ICC (good) values below the excellence values, although they had a low SEM, which also suggests excellent reliability. The same happens in most validation studies of this questionnaire, in which vigorous-intensity activities and sports activities had lower ICC values, which may be related to the reduced sample size for these higher-intensity activities [15, 23, 24, 27].

As for criterion validity, the correlation obtained for total PA, in the original study, between the PPAQ and the accelerometer, was 0.27, classified as a weak correlation [15]. In the present study, this value was -0.030, in turn, classified as weak and negative. In other studies [15, 16, 20, 21, 23, 24], the correlation was classified between weak to moderate (0.201 to 0.35) [21, 24], considering that there is no criterion validity, with different gold measurement instruments used. The accelerometer was used in the original study and in the validation study for the Chinese population [15, 24]. The pedometer, a measure used only to estimate the number of steps taken, was used in Vietnam and Turkey [16, 19, 20]. A multi-sensor monitor, Sensewear Mini Armband (SWA; BodyMedia Inc., Pittsburgh, USA) was used to assess physical activity and energy expenditure. This monitor provides a more accurate estimation of energy expenditure than accelerometry-based devices [20] and has shown a good correlation with indirect calorimetry measured on pregnant women [16]. As an exception, in the validation study for the Turkish population, moderate correlation values (0.672) were obtained between the PPAQ and the IPAQ, and a strong correlation between the PPAQ and the pedometer (0.700) [20]. In a 2011 systematic review, which showed the correlations obtained for total PA, in various countries, between the IPAQ and an objective measure such as an actometer, accelerometer, or pedometer, this value ranged between 0.09 and 0.39. All these results obtained, both for the study of the IPAQ and the PPAQ, show that correlating a subjective measure with an objective one reveals values without significance and, therefore, there is no criterion validity [43].

In view of the above, in the validation studies carried out in different countries, three methods of physical assessment were used: the accelerometer, the pedometer, and the SWA.

The most used is the ActiGraph GTX3 accelerometer [8]. This is placed on the anterosuperior iliac crest of the participant’s dominant limb, and, throughout pregnancy, there is an increase in abdominal volume [44]. That may affect the positioning of the accelerometer, causing an inclination of the device and, thus, influence the quality of the data collected [45]. This inclination is aggravated towards the end of pregnancy. On the other hand, accelerometers are unable to measure certain types of activities that involve upper torso movement, aquatic activities, and stationary cycling [8, 15, 22, 24], which can lead to an underreporting of activity as women perform various domestic activities without lower limb movement, such as cooking, ironing, bathing their children, etc. However, the use of this instrument for 7 days could lead to greater awareness of PA practice, leading these women to modify their behavior, as they are being studied [15, 23].

Questionnaires stand out for being more accessible instruments for the assessment of PA and, therefore, important instruments in epidemiological studies. However, studies report that questionnaires tend to overestimate the prevalence of PA during pregnancy [46]. This may be due to information and memory bias [47], as pregnant women may not remember exactly how much time they need to perform different activities, particularly in household activities, when they may perform double tasks, for example, cooking while bathing the children, what can lead to distortion and limitation of the study results.

In addition, PPAQ calculations were based on values from the compendium, which in turn are based on data from non-pregnant men and women. In addition to this limitation, most of the activity classifications referred to in the compendium are derived from laboratory and subjective judgment data and, as such, may not accurately reflect energy expenditure [15, 16]. Thus, the energy calculation of the participants may not correspond to reality, what could directly influence the results obtained by the correlation between the PPAQ and the accelerometer. This is because the questionnaire measures the pregnant woman’s perception of the level of PA that she practices, while the biophysical measures (accelerometer) measure behaviors (number of steps; metabolic expenses; counts). If the pregnant woman is aware of the PA that she practices, it will not change her behavior; hence, that is the relevance of measuring perceptions and measuring PA with biophysical measures, as both provide important and complementary information. In addition, the questionnaire gives us information on what activities pregnant women practice, which facilitates, for example, counseling and planning to promote PA.

An interesting fact observed in our study population was that we had a floor effect with about 50% of the questions with the chosen answer option “none”, and we believe that this is justified by the characteristics of the sample. Six questions are related to childcare, and in our sample, 50% of women were primiparous. Another question is related to the care of the elderly and 2 others with animals, leading us to believe that part of these answers may be related to the fact that these women do not have animals or elderly people in their care.

Other issues identified are related to vigorous exercise (running, climbing ramps, dancing, swimming…) which, according to the physical activity reported by women, more than half do not practice structured physical exercise or do vigorous activities. This information, despite being little discriminatory in statistical terms, could modify the discriminatory power of the instrument and presents important information about the exact type of task performed by pregnant women in their daily routine.

We consider some points in favor of our study: the large sample size; the heterogeneity of the sample, since the sample collection sites belong to the public service, encompassing pregnant women with different socioeconomic levels and lifestyles; the existence of two open-ended questions, giving pregnant women the opportunity to report more activities that they practice that are not mentioned in the questionnaire.

## Conclusion

The PPAQ questionnaire is translated and adapted for the Portuguese population and has excellent reliability. As for validity, it presents content validity and weak criterion validity. However, a version was obtained that can be used by health professionals as an instrument to assess the physical activity of pregnant women in Portugal.

## Supporting information

S1 Appendix Questionnaire-Inclusivity in global research: Inclusivity in global research. Additional information regarding the ethical, cultural, and scientific considerations specific to inclusivity in global research is included in the Supporting information.
(PDF)

S2 Appendix Translated version of the questionnaire adapted to Portuguese. (DOC)

S1 Data (XLSX)
