# Supplementary material for: Cultural adaptation and validation of the “Pregnancy Physical Activity Questionnaire” for the Portuguese population
Source: PLoS One. 2023 Jan 10;18(1):e0279124. doi: 10.1371/journal.pone.0279124 (PMC9831324; doi:10.1371/journal.pone.0279124)
Supplement: S2 Appendix — (DOC) [file pone.0279124.s002.doc]

Supplement Portuguese - Changes at the semantic level.

| **Question number** | **Original version** | **Final version after changes** |
| --- | --- | --- |
| **8** | (…) a brincar com crianças enquanto está a caminhar ou a correr | (…) a brincar com crianças enquanto está a andar ou a correr |
| **9** | (…) a transportar crianças | (…) a pegar em crianças ao colo |
| **11** | (…) sentada e a usar computador ou a escrever, mas sem estar a trabalhar | (…) sentada a usar computador ou a escrever, sem estar no local de trabalho (emprego) |
| **13** | (…) sentada a ler, a falar, ou ao telefone, mas sem estar a trabalhar | (…) sentada a ler, a falar, ou ao telefone,  sem estar no local de trabalho (emprego) |
| **17** | (…) a limpezas mais pesadas (aspirar, passar o chão, varrer, lavar janelas) | (…) a limpezas mais pesadas (aspirar, limpar o chão com esfregona, varrer, lavar janelas) |
| **18** | (…) a cortar relva com uma máquina automática | (…) a cortar relva com uma máquina automática em que esteja sentada |
| **19** | (…) a cortar relva com uma máquina manual, a apanhar ervas, a jardinar | (…) jardinar a apanhar ervas ou a cortar relva com uma máquina em que tenha que andar |
| **25** | (…) a subir rapidamente montes, por prazer ou exercício | (…) a andar rapidamente em terrenos com inclinação (a subir) por prazer ou exercício |
| **26** | (…) a praticar *jogging* | (…) a correr |
| **27** | (…) a praticar em exercícios pré-natal | (…) a realizar exercício em aulas de preparação para o parto |
| **28** | (…) a praticar natação | (…) a nadar |
| **33** | (…) a estar de pé ou a caminhar lentamente transportando objetos (de peso superior a um recipiente de leite de 1 gal. (3,78 lts.)) | (…) a estar de pé ou andar lentamente no local de trabalho transportando objectos (de peso igual ou superior a um garrafão de água (5 Litros)) |
| **35** | (…) a caminhar rapidamente, a transportar objetos (de peso superior a um recipiente de leite de 1 gal. (3,78 lts.)) | (…) a andar rapidamente, no local de trabalho transportando objectos (de peso igual ou superior a um garrafão de água (5 Litros)) |

Supplement English - Changes at the semantic level.

| **Question number** | **Original version** | **Final version after changes** |
| --- | --- | --- |
| **8** | (…) playing with children while walking or running | (…) playing with children while walking or running |
| **9** | (…) transporting children | (…) to take children in the arm |
| **11** | (…) sitting and using a computer or writing, but not working | (…) sitting using a computer or writing, not at work (employment) |
| **13** | (…) sitting reading, talking, or on the phone, but not working | (…) sitting reading, talking, or on the phone,  not being at work (job) |
| **17** | (…) to heavier cleaning (vacuuming, mopping, sweeping, washing windows) | (…) to heavier cleaning (vacuuming, mopping the floor, sweeping, washing windows) |
| **18** | (…) mowing grass with an automatic machine | (…) mowing grass with an automatic seated machine |
| **19** | (…) mowing the lawn with a manual machine, picking weeds, gardening | (…) gardening, picking weeds, or cutting grass with a machine that has to walk |
| **25** | (…) to quickly climb hills, for pleasure or exercise | (…) to walk quickly on sloping (uphill) terrain for pleasure or exercise |
| **26** | (…) jogging | (…) to run |
| **27** | (…) to practice prenatal exercises | (…)to exercise in childbirth preparation classes |
| **28** | (…) practicing swimming | (…) to swim |
| **33** | (…) standing or walking slowly carrying objects (weighing more than 1 gal. (3,78 lts.)) | (…) standing or walking slowly in the workplace carrying objects (weight equal to or greater than a gallon of water (5 Liters)) |
| **35** | (…) walking quickly, carrying objects (weighing more than a 1-gallon milk container (3.78 lt.)) | (…) walking quickly, in the workplace carrying objects (weight equal to or greater than a gallon of water (5 Liters)) |
